# Microenvironment at a Distance: Multi-Endocrine-Organ Radiomics to Identify Systemic Signatures in PSMA-Negative Prostate Cancer

**DOI:** 10.3390/cancers18111767

**Published:** 2026-05-28

**Authors:** Hamid Abdollahi, Sara Harsini, Fereshteh Yousefirizi, Bahareh Hatami, François Bénard, Ahmad Shariftabrizi, Ian Alberts, Arman Rahmim

**Affiliations:** 1 Department of Radiology, University of British Columbia, Vancouver, BC V5Z 1M9, Canada; habdollahi@bccrc.ca (H.A.);; 2 Department of Basic and Translational Research, BC Cancer Research Institute, 675 West 10th Ave., Vancouver, BC V5Z 1L3, Canada; 3 Department of Medical Imaging, University of Toronto, Toronto, ON M5T 1W7, Canada; 4 Department of Radiology, Tehran University of Medical Sciences (TUMS), Tehran 1416753955, Iran; 5 Division of Nuclear Medicine, Department of Radiology, University of Iowa Carver College of Medicine, Iowa City, IA 52242, USA; 6 Research Center for Molecular and Cellular Imaging (RCMCI), Advanced Medical Technologies and Equipment Institute (AMTEI), Tehran University of Medical Sciences (TUMS), Tehran 1416753955, Iran; 7 Departments of Physics & Biomedical Engineering, University of British Columbia, Vancouver, BC V6T 1Z1, Canada

**Keywords:** PSMA-negative prostate cancer, radiomics, PET/CT, endocrine system, clinical progression

## Abstract

**Background/Introduction**: Prostate cancer (PCa) is the most commonly diagnosed malignancy among men and remains a major cause of cancer-related mortality worldwide. We aimed to evaluate whether radiomic features extracted from normal endocrine organs, combined with clinical variables, could predict clinical progression in patients with PSMA-negative prostate cancer. **Materials and Methods**: In this retrospective study, 101 men with biochemically recurrent prostate cancer and negative [^18^F]DCFPyL PET/CT scans were included. Radiomic features were extracted from the adrenal glands, thyroid, the hypothalamus–pituitary complex, and testes. Post-imaging variables were excluded to prevent temporal data leakage. Models were developed using a stratified train/test split framework with preprocessing and feature selection performed exclusively within the training subset prior to evaluation on the held-out test set. Performance was evaluated using AUC, accuracy, sensitivity, specificity, and Brier score, while bootstrap confidence intervals and DeLong analysis were used for statistical assessment. **Results**: Multimodal fusion models integrating CT radiomics, PET radiomics, and clinical variables demonstrated the strongest predictive performance. The highest-performing model combined TESTIS_CT and TESTIS_PET radiomics with clinical variables, achieving an AUC of 0.758 (95% CI: 0.653–0.849). Clinical-only models remained highly competitive, with the best configuration achieving an AUC of 0.727 (95% CI: 0.618–0.833). PET + clinical and CT + clinical models achieved AUC values of up to 0.733 and 0.729, respectively, while imaging-only models demonstrated substantially lower discrimination. Although endocrine organ radiomics numerically improved predictive performance and specificity, DeLong analysis demonstrated no statistically significant improvement beyond clinical variables alone. **Discussion**: These findings suggest that endocrine organ radiomics may provide complementary system-level imaging biomarkers reflecting tumor–host interactions in PSMA-negative prostate cancer. However, their incremental clinical value remains modest. **Conclusions**: Endocrine organ radiomics combined with clinical variables demonstrated promising predictive performance in PSMA-negative prostate cancer, particularly in multimodal fusion models. Nevertheless, the added value beyond clinical variables alone was not statistically significant and requires validation in larger independent cohorts.

## Simple Summary

This study evaluated endocrine organ radiomics from [^18^F]DCFPyL PET/CT for predicting clinical progression in PSMA-negative prostate cancer. Multimodal models integrating CT, PET, and clinical variables achieved the best predictive performance, although clinical-only models remained highly competitive. Endocrine organ radiomics demonstrated complementary but modest predictive value beyond conventional clinical variables, supporting their exploratory role as potential system-level biomarkers of tumor–host interaction.

## 1. Introduction

Prostate cancer (PCa) is the most commonly diagnosed malignancy in men and remains a major contributor to cancer-related mortality worldwide [1]. Its biological behavior is highly heterogeneous, ranging from indolent disease to aggressive metastatic spread involving bone, lymph nodes, and visceral organs [2]. PSMA-targeted PET/CT has markedly enhanced diagnostic accuracy by enabling sensitive detection of both primary and metastatic lesions, even at low PSA levels [3]. However, a subset of patients exhibit PSMA-negative PET/CT findings despite clinical progression [4,5], revealing a disconnect between tumor activity and tracer uptake. This highlights a biological gap in lesion-based imaging and underscores the need for systemic biomarkers capable of detecting recurrence when conventional approaches fail. This limitation further suggests that clinically relevant disease biology may extend beyond visually detectable tumor lesions and may instead involve broader systemic alterations not captured by conventional lesion-centered imaging approaches.

Clinical outcomes in PCa are shaped not only by tumor characteristics but also by systemic host factors, including hormonal, metabolic, and immune regulation [6,7]. Although androgen deprivation therapy and next-generation antiandrogens have improved survival, many patients with advanced or PSMA-negative disease continue to relapse [8,9], reflecting complex tumor–host interactions [10]. Identifying imaging correlates of these systemic processes may improve prognostication and guide precision treatment. In particular, endocrine and neurohormonal pathways may influence tumor progression, treatment resistance, and systemic adaptation, making endocrine-related imaging biomarkers an area of growing interest in precision oncology.

Radiomics provides a non-invasive approach to quantify tissue heterogeneity from standard imaging [11]. In PCa, radiomics derived from PSMA-PET and CT has shown potential in tumor characterization, recurrence prediction, and treatment response assessment [12,13], yet prior studies largely focused on tumor regions, neglecting the physiological information contained in normal organs. Evidence from other cancers suggests that radiomic features from uninvolved organs can reflect systemic effects of malignancy [14,15]. Such findings support the concept that cancer-related imaging phenotypes may emerge not only from tumors themselves, but also from host organs involved in systemic metabolic, endocrine, and inflammatory regulation.

Given the central hormonal and metabolic roles of endocrine organs, including the adrenal glands, thyroid, the hypothalamus–pituitary complex, and testes, subtle imaging changes within these structures may serve as biomarkers of systemic disease activity. Accordingly, we propose a multi-organ radiomics framework that integrates quantitative features from [^18^F]DCFPyL PSMA-PET/CT with clinical parameters to predict clinical progression in PSMA-negative PCa, aiming to identify endocrine-based imaging signatures of tumor–host interactions. To our knowledge, this represents one of the first studies to investigate endocrine organ radiomics in PSMA-negative prostate cancer using a multi-organ PET/CT-based framework focused on systemic disease characterization and progression prediction.

## 2. Methods and Materials

**Figure 1:**
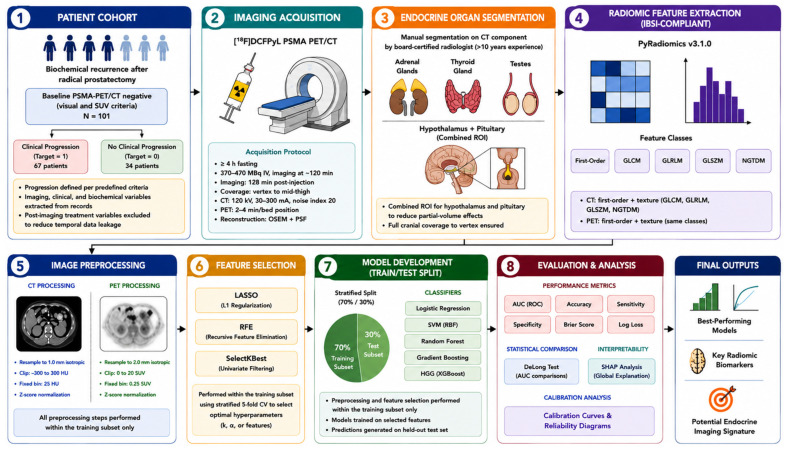
Overview of the endocrine organ radiomics framework for predicting clinical progression in PSMA-negative prostate cancer. The workflow included patient cohort selection, [^18^F]DCFPyL PET/CT acquisition, endocrine organ segmentation, IBSI-compliant radiomic feature extraction, standardized image preprocessing, feature selection, machine learning model development using a stratified train/test split framework, performance evaluation, calibration analysis, DeLong statistical comparison, and SHAP-based interpretability analysis to identify complementary endocrine organ imaging biomarkers and multimodal predictive signatures.

The flowchart of the study is presented in Figure 1, with the methodological workflow illustrated in detail. The following sections provide a comprehensive description of the study methods.

### 2.1. Patient Dataset

This study used the 101-patient cohort originally reported by Harsini et al. [5]. All patients had biochemical recurrence following radical prostatectomy and underwent a baseline [^18^F]DCFPyL PET/CT scan that was negative according to both visual assessment and SUV-based criteria. The median PSA level at the time of imaging was 0.56 ng/mL (range: 0.4–11.3). Clinical progression (CP) was defined as the radiologic emergence of new lesions on follow-up imaging, including PET/CT, MRI, CT, or bone scan; PSA-only progression was not considered CP. Based on this definition, 67 patients experienced clinical progression during follow-up, while 34 patients remained progression-free. Although 36 patients (36%) received salvage radiotherapy (sRT) within 3 months after imaging, all models were designed to use only baseline variables available at the time of imaging. Accordingly, post-imaging treatment variables (e.g., RT after PET, dose-related parameters) were excluded from all predictive models to prevent temporal data leakage and ensure clinical applicability.

### 2.2. PET/CT Imaging Protocol

Patients fasted for 4 h before imaging. They received an intravenous injection of 237–474 MBq [^18^F]DCFPyL, adjusted by body weight with ±10% target activity variation. Imaging was performed 120 min post-injection from the vertex/top of head to mid-thigh/proximal femurs using a Discovery PET/CT 600 or 690 scanner (GE Healthcare). A non-contrast CT was first acquired for localization and attenuation correction using 120 kV, automatic mA selection 30–200 mA, and noise index 20. PET images were then acquired at 2–4 min per bed position, adjusted for patient girth, and reconstructed using ordered subset expectation maximization with point spread function modeling.

### 2.3. Image Segmentation

Normal endocrine organs were segmented manually on the CT component of each PET/CT scan. The adrenal glands, thyroid, and testes were delineated individually due to their clear anatomical boundaries. Given the limitations of low-dose CT in resolving central brain structures, the hypothalamus and pituitary gland were segmented as a single combined region of interest to reduce partial-volume effects and improve feature stability. All segmentations were performed by a board-certified radiologist with >10 years of oncologic imaging experience, ensuring consistency and anatomical accuracy. Additionally, all PET/CT scans included full cranial coverage up to the vertex, confirming that hypothalamic sellar structures were fully captured and not truncated at the skull base.

### 2.4. Radiomic Feature Extraction

Radiomic features were extracted from attenuation-corrected PET and CT volumes following Image Biomarker Standardization Initiative (IBSI) recommendations. First-order intensity metrics and second-order texture matrices (GLCM, GLRLM, GLSZM, NGTDM) were computed using the PyRadiomics v3.1.0 library, which adheres to standardized IBSI feature definitions [16].

### 2.5. Image Preprocessing

To maximize feature reproducibility, CT volumes were resampled to 1.0 mm isotropic spacing and PET volumes to 2.0 mm spacing. CT intensities were clipped to –500 to 500 HU, and PET intensities were limited to 0–30 SUV. Fixed-bin discretization (25 HU for CT; 0.25 SUV for PET) was applied, and PET images were re-segmented to exclude negative values. All preprocessing steps were standardized across the cohort.

### 2.6. Data Preprocessing

Clinical, CT-radiomic, and PET-radiomic features were merged by patient identifier. Clinical variables that could introduce temporal leakage, including RT after PET, ADT after PET, and dose-related variables, were removed before model development. Missing values were handled using median imputation, chosen for its robustness in small datasets and reduced sensitivity to outliers. Near-zero variance features were removed using variance thresholding. Highly correlated features were filtered using a Pearson correlation threshold of $\left|r\right|>0.85$, retaining only nonredundant predictors. Continuous features were then standardized using z-score normalization.

To prevent information leakage, all preprocessing steps, including median imputation, variance filtering, correlation filtering, and feature scaling, were performed exclusively within the training subset and then applied to the corresponding validation fold.

### 2.7. Feature Selection

Feature selection was performed independently within the training subset using three predefined methods: ANOVA F-test with SelectKBest, LASSO-based selection using L1-regularized logistic regression with internal cross-validation, and recursive feature elimination using class-weighted logistic regression. The number of selected features was fixed at k = 5 for all models and configurations to reduce model selection bias and improve comparability across experiments. Selected features were recorded to assess feature selection stability across model configuration.

### 2.8. Model Development

Five machine learning classifiers were evaluated: logistic regression, random forest, gradient boosting, support vector machine with a radial basis function kernel, and histogram-based gradient boosting. Logistic regression and support vector machine models use class weighting to account for class imbalance. Random forest uses balanced subsampling. All models were trained using only the selected features from the training subset.

Model configurations included clinical-only, CT-only, PET-only, CT + clinical, PET + clinical, multi-organ CT-only, multi-organ PET-only, true CT + PET fusion, and CT + PET + clinical fusion models. Endocrine organ radiomic inputs included the adrenal glands, hypothalamus–pituitary (Hyp-Pit) complex, testes, and thyroid.

### 2.9. Validation Strategy

Model performance was evaluated using a stratified train/test split framework with 70% training data and 30% held-out testing data. All preprocessing operations, including median imputation, feature scaling, and feature selection, were performed exclusively within the training subset prior to application to the independent test set to reduce potential information leakage. Final performance metrics, bootstrap confidence intervals, calibration analysis, and DeLong statistical comparisons were calculated using held-out test set predictions.

### 2.10. Performance Evaluation and Statistical Analysis

The primary performance metric was the area under the receiver operating characteristic curve (AUC). Secondary metrics included accuracy, sensitivity, specificity, precision, F1-score, Brier score, and log loss. Binary predictions were generated using predicted probabilities and standard classification thresholds. Uncertainty was estimated using bootstrap resampling with 1000 iterations, generating 95% confidence intervals for AUC, accuracy, sensitivity, specificity, precision, F1-score, Brier score, and log loss. Statistical comparison between candidate models and the best clinical-only baseline model was performed using the DeLong test for correlated ROC curves. The clinical-only model was used as the reference comparator.

### 2.11. Calibration Analysis

Model calibration was assessed using Brier score, log loss, and calibration plots. Calibration curves compared the mean predicted probability with the observed event frequency across probability bins. ROC and calibration plots were generated for the top-performing models.

### 2.12. Feature Stability and Interpretability

Feature selection stability was assessed by recording the selected features in the held-out test set validation and counting their selection frequency across model configurations. For the final best-performing model, feature importance was estimated using model-specific importance values when available, coefficient magnitudes for linear models, or permutation importance when intrinsic feature importance was not available. SHAP (SHapley Additive exPlanations) analysis was performed for representative top-performing CT-based, PET-based, clinical-only, and multimodal fusion models to evaluate feature importance and model interpretability.

### 2.13. Noise and Signal Considerations

Because normal endocrine tissues may have low physiologic PSMA uptake, the PET signal in these structures may be influenced by blood pool activity and image noise. Low-dose CT also provides limited soft-tissue contrast, particularly for small structures such as the Hyp-Pit region. To reduce noise-related bias, standardized preprocessing, variance filtering, correlation filtering, fold-specific feature selection, and out-of-fold validation were applied. Nevertheless, the analysis remains exploratory, and the absence of external validation, endocrine laboratory biomarkers, and segmentation reproducibility testing limits biological interpretation.

## 3. Results

### 3.1. Overall Modeling Results

Clinical-only models demonstrated robust and reproducible predictive capability across multiple machine learning algorithms and feature selection strategies. The strongest clinical-only configuration used recursive feature elimination (RFE) with gradient boosting and achieved an AUC of 0.727 (95% CI: 0.618–0.833), accuracy of 0.714, sensitivity of 0.792, specificity of 0.605, Brier score of 0.241, and log loss of 0.908. Alternative clinical-only configurations demonstrated slightly lower performance, including SelectKBest with histogram-based gradient boosting (HGB) and LASSO with gradient boosting. Overall, feature selection analysis demonstrated that several conventional clinicopathologic variables, including tumor stage, nodal status, lesion location, Gleason score, PSA-associated biomarkers, and baseline treatment category, were consistently retained across predictive pipelines, highlighting their substantial contribution within the final framework.

Among CT-only pipelines, TESTIS_CT demonstrated the strongest standalone CT performance, achieving an AUC of 0.636 (95% CI: 0.515–0.746), while HYPO_PIT_CT demonstrated relatively high sensitivity despite lower overall discrimination (Table 1). Integration of CT radiomics with clinical variables substantially improved predictive performance. The strongest CT + clinical model combined TESTIS_CT radiomics with SelectKBest and support vector machine (SVM), achieving an AUC of 0.729 (95% CI: 0.619–0.834), accuracy of 0.670, sensitivity of 0.623, specificity of 0.737, Brier score of 0.206, and log loss of 0.604. HYPO_PIT_CT + clinical also demonstrated strong performance using RFE and HGB (AUC: 0.709), whereas ADRENAL_CT + clinical demonstrated relatively high specificity (0.816). Multi-organ CT-only combinations did not consistently improve predictive performance compared with the strongest single-organ CT models, with AUC values generally remaining between approximately 0.60 and 0.62 (Table 2).

PET-only models generally demonstrated higher sensitivity than CT-only pipelines. The strongest PET-only configuration was THYROID_PET using RFE and HGB, which achieved an AUC of 0.710 (95% CI: 0.587–0.827), accuracy of 0.714, sensitivity of 0.868, specificity of 0.500, Brier score of 0.219, and log loss of 0.670 (Table 1). Integration of PET radiomics with clinical variables further improved discrimination performance. The strongest PET + clinical model combined TESTIS_PET radiomics with SelectKBest and HGB, achieving an AUC of 0.733 (95% CI: 0.630–0.826), accuracy of 0.692, sensitivity of 0.679, specificity of 0.711, Brier score of 0.221, and log loss of 0.679. HYPO_PIT_PET + clinical demonstrated comparable performance with an AUC of 0.726, while ADRENAL_PET + clinical achieved relatively high specificity (0.789). Multi-organ PET-only combinations demonstrated only modest improvement over single-organ PET models, with the strongest multi-organ PET-only configuration achieving an AUC of 0.678.

Fusion models integrating CT radiomics, PET radiomics, and clinical variables consistently produced the strongest overall predictive performance (Table 2). The highest-performing overall model combined TESTIS_CT and TESTIS_PET radiomics with clinical variables using RFE and gradient boosting, achieving an AUC of 0.758 (95% CI: 0.653–0.849), accuracy of 0.703, sensitivity of 0.604, specificity of 0.842, Brier score of 0.254, and log loss of 0.995. A nearly identical HGB-based configuration achieved an AUC of 0.757 while improving sensitivity to 0.792 and overall accuracy to 0.747. Random forest also demonstrated strong and balanced fusion performance, achieving an AUC of 0.751 and the lowest Brier score (0.208) among the strongest multimodal pipelines (Table 3). In contrast, true CT + PET fusion without clinical variables demonstrated substantially lower discrimination, with TESTIS_CT + TESTIS_PET fusion without clinical integration achieving an AUC of only 0.636. These findings emphasize the major contribution of conventional clinical information within the final predictive framework.

Among machine learning algorithms, gradient boosting and HGB consistently produced the strongest overall performance, particularly within multimodal fusion settings (Table 3). SVM demonstrated strong performance in CT + clinical models, while logistic regression generally demonstrated lower discrimination but remained competitive in selected PET-based and multi-organ pipelines. Regarding feature selection strategies, RFE consistently generated the strongest and most stable models across multimodal fusion settings, achieving the highest overall AUC of 0.758. LASSO achieved a maximum AUC of 0.742, while SelectKBest achieved 0.733 (Table 4).

### 3.2. SHAP, Feature Importance, and Radiomic Pattern Analysis

**Figure 2:**
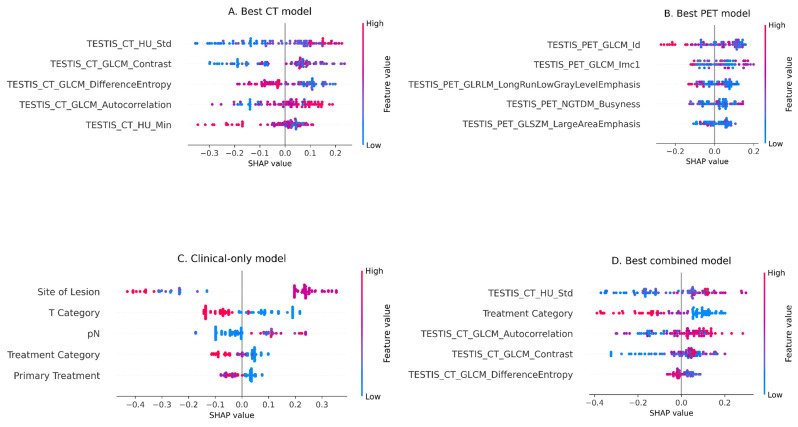
SHAP beeswarm plots for the strongest (**A**) CT-based, (**B**) PET-based, (**C**) Clinical-only, and (**D**) Multimodal fusion models. The plots illustrate the relative contribution of the most influential features to model predictions. Positive SHAP values indicate increased contribution toward the predicted outcome probability, whereas negative values indicate reduced contribution. Feature values are represented by the color scale ranging from low (blue) to high (pink).

SHAP beeswarm analysis demonstrated distinct predictive patterns across CT, PET, clinical, and multimodal fusion models (Figure 2). In the strongest CT-based model (Figure 2A), TESTIS_CT-derived heterogeneity biomarkers including HU_Std, GLCM_Contrast, DifferenceEntropy, and GLCM_Autocorrelation demonstrated the strongest predictive contributions. The strongest PET-based model (Figure 2B) was dominated by PET texture biomarkers including GLCM_Id, GLCM_Imc1, GLRLM_LongRunLowGrayLevelEmphasis, and NGTDM_Busyness, highlighting the importance of PET uptake heterogeneity and metabolic nonuniformity. Clinical-only SHAP analysis (Figure 2C) identified Treatment Category, T Category, pN stage, Site of Lesion, and Primary Treatment as dominant non-imaging predictors. In the multimodal fusion model (Figure 2D), both radiomic and clinical biomarkers contributed jointly, with TESTIS_CT_HU_Std, TESTIS_CT_GLCM_Autocorrelation, TESTIS_CT_GLCM_Contrast, Treatment Category, and T Category demonstrating strong predictive impact.

Overall, the strongest predictive biomarkers were predominantly related to texture heterogeneity, entropy, gray-level nonuniformity, and metabolic variability. CT-derived predictors mainly reflected texture complexity, HU variability, and local heterogeneity, whereas PET-derived biomarkers were related to metabolic uptake heterogeneity and intensity nonuniformity. TESTIS-derived radiomics consistently demonstrated strong predictive relevance across both standalone and multimodal fusion pipelines, suggesting biologically informative endocrine-associated imaging phenotypes related to clinical progression in PSMA-negative prostate cancer.

### 3.3. Calibration Analysis

**Figure 3:**
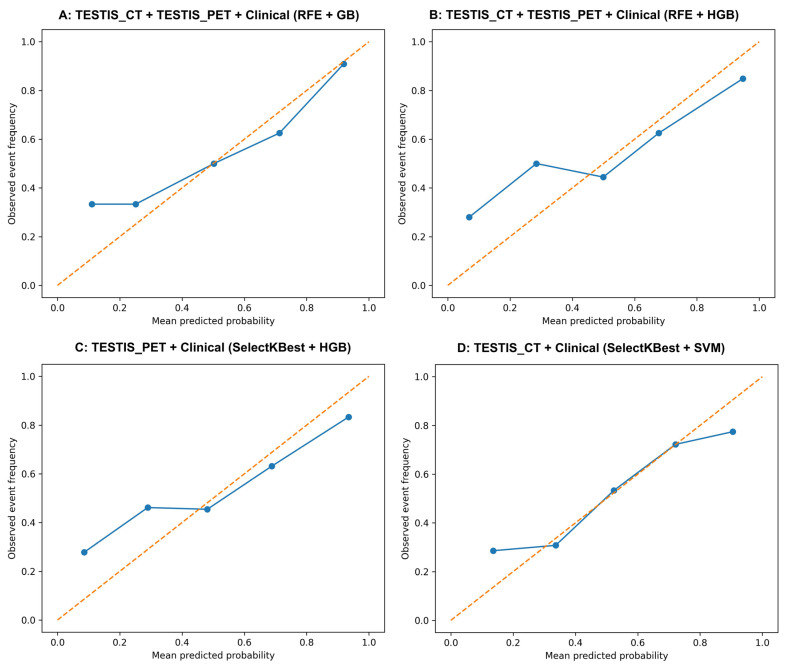
Calibration curves of the best-performing machine learning models, including multimodal fusion, PET + clinical, and CT + clinical configurations. Panels show TESTIS_CT + TESTIS_PET + clinical fusion using RFE + gradient boosting (**A**) and RFE + HGB (**B**), TESTIS_PET + clinical using SelectKBest + HGB (**C**), and TESTIS_CT + clinical using SelectKBest + SVM (**D**). GB: gradient boosting; HGB: histogram-based gradient boosting; SVM: support vector machine.

Calibration analysis demonstrated moderate agreement between predicted probabilities and observed event frequencies across the strongest multimodal fusion, PET + clinical, and CT + clinical models (Figure 3). The calibration curves showed relatively stable agreement with the ideal reference line, particularly within intermediate and higher probability ranges. Among the evaluated models, the TESTIS_CT + TESTIS_PET + clinical fusion models demonstrated the most consistent calibration behavior, while the PET + clinical and CT + clinical configurations also showed acceptable probabilistic reliability. These findings were further supported by the favorable Brier score and log loss values observed across the top-performing models.

### 3.4. DeLong Statistical Comparison

**Figure 4:**
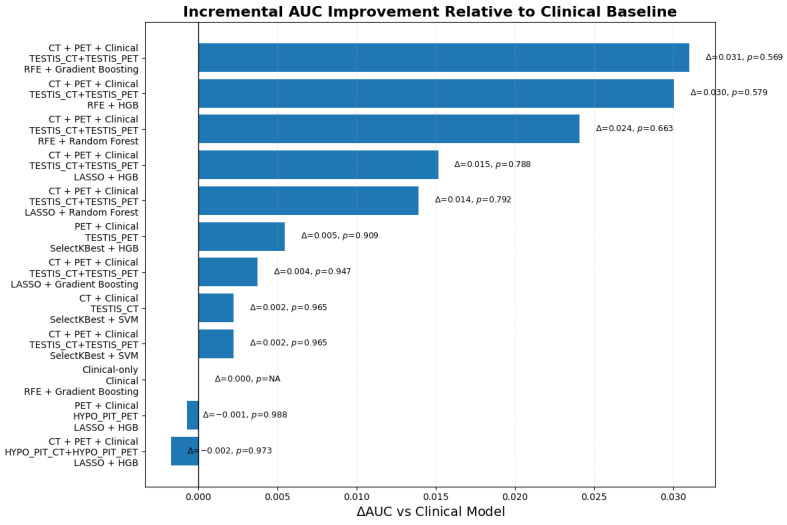
Incremental AUC improvement (ΔAUC) of CT, PET, and multimodal fusion models relative to the clinical-only baseline using DeLong statistical comparison. Although several multimodal fusion pipelines demonstrated numerical improvement in discrimination performance, none achieved statistically significant AUC improvement (all *p* > 0.05). The largest improvement was observed for the TESTIS_CT + TESTIS_PET + clinical fusion model using RFE and Gradient Boosting (ΔAUC = 0.031) (Table 1). Performance of the strongest single-organ CT- and PET-based models with and without clinical integration.

DeLong comparison analysis demonstrated that multimodal fusion models provided only limited incremental improvement in discrimination performance relative to the clinical-only baseline (Figure 4). The largest improvement was observed for the TESTIS_CT + TESTIS_PET + clinical fusion model using RFE and gradient boosting, with a ΔAUC of 0.031 (*p* = 0.569). Similar modest numerical improvements were observed for fusion models using histogram-based gradient boosting (ΔAUC = 0.030; *p* = 0.579) and random forest (ΔAUC = 0.024; *p* = 0.663). PET + clinical and CT + clinical models demonstrated smaller gains, with ΔAUC values ranging from 0.002 to 0.015. However, none of the evaluated models demonstrated statistically significant improvement over the clinical-only baseline (all *p* > 0.05). Overall, these findings suggest that endocrine organ radiomics provided complementary but relatively modest additional predictive value beyond conventional clinical variables.

## 4. Discussion

This study demonstrates that radiomic profiling of normal endocrine organs may reveal systemic imaging signatures related to clinical progression in men with PSMA-negative prostate cancer. By integrating CT- and PET-derived features from the adrenal glands, Hyp-Pit complex, thyroid, and testes with clinical variables, we observed that structurally normal but hormonally active organs carry quantifiable textural patterns relevant to disease behavior and systemic tumor–host interactions [14,15,17]. The strongest multimodal fusion model, integrating TESTIS_CT and TESTIS_PET radiomics with clinical variables using RFE and gradient boosting, achieved an AUC of 0.758 (95% CI: 0.653–0.849), while the best clinical-only model achieved an AUC of 0.727 (95% CI: 0.618–0.833). Although multimodal integration numerically improved discrimination performance and specificity, the incremental improvement relative to the clinical-only baseline remained modest (ΔAUC = 0.031) and did not reach statistical significance in DeLong analysis (*p* = 0.569). Therefore, these findings should be interpreted as exploratory and hypothesis-generating rather than confirmatory. Calibration analysis also demonstrated moderate agreement between predicted probabilities and observed outcomes across the strongest multimodal fusion, PET + clinical, and CT + clinical models, although these findings should be interpreted cautiously given the limited cohort size and absence of external validation.

Among the evaluated endocrine organs, the adrenal glands demonstrated distinct CT- and PET-based radiomic patterns related to clinical progression. CT-derived biomarkers such as first-order kurtosis, HU standard deviation, and GLSZM Zone Entropy were frequently identified in predictive pipelines, suggesting structural heterogeneity and variable tissue organization, while PET-derived features including GLSZM_SizeZoneNonUniformityNormalized and NGTDM_Busyness reflected metabolic heterogeneity and irregular uptake patterns. In the final analysis, ADRENAL_CT + clinical models achieved an AUC of 0.700 (95% CI: 0.590–0.809), whereas ADRENAL_PET + clinical models achieved an AUC of 0.695 (95% CI: 0.593–0.798), both demonstrating relatively high specificity values (0.816 and 0.789, respectively). Although adrenal-only imaging models demonstrated more limited discrimination performance, integration with clinical variables improved overall model balance and stability. Biologically, these findings may reflect adrenal cortical remodeling and endocrine adaptation related to chronic hypothalamic–pituitary–adrenal activation and sustained androgen synthesis. Previous translational studies have demonstrated that, even after castration, the adrenal glands continue to produce androgen precursors such as DHEA and androstenedione through CYP17A-mediated pathways, potentially contributing to persistent androgen receptor signaling and castration-resistant progression [18,19]. Accordingly, increased radiomic heterogeneity within the adrenal glands may reflect subtle systemic hormonal remodeling rather than direct tumor involvement. Nevertheless, these biological interpretations remain exploratory, and future studies incorporating endocrine laboratory biomarkers such as cortisol, ACTH, and DHEA-S will be necessary to validate whether adrenal radiomic phenotypes truly reflect HPA axis activity and endocrine adaptation in PSMA-negative prostate cancer.

The hypothalamus–pituitary complex also emerged as an important contributor within several predictive pipelines, particularly when integrated with clinical variables. CT- and PET-derived features such as GLCM Autocorrelation, Idmn, and NGTDM Contrast, which reflect spatial regularity, texture organization, and metabolic heterogeneity, were repeatedly identified among higher-performing models. In the final analysis, HYPO_PIT_CT + clinical models achieved an AUC of 0.709 (95% CI: 0.591–0.813), while HYPO_PIT_PET + clinical models achieved an AUC of 0.726 (95% CI: 0.616–0.830), demonstrating relatively balanced sensitivity and specificity profiles. These findings suggest that radiomic alterations within the Hyp-Pit region may reflect systemic endocrine adaptation related to androgen suppression and disease progression. Chronic GnRH and LH feedback during androgen deprivation therapy has been related to pituitary remodeling and altered vascularity, which may contribute to subtle imaging heterogeneity potentially detectable through radiomic analysis [2,20]. When combined with PSA kinetics and treatment-related variables, these imaging signatures may represent indirect markers of endocrine axis adaptation or treatment-related physiologic stress rather than direct tumor-related changes. Nevertheless, the biological interpretation of these findings remains exploratory, and future validation incorporating LH, FSH, GnRH analog treatment data, endocrine biomarkers, and dedicated pituitary MRI correlation will be required to determine whether these radiomic signatures truly reflect hypothalamic–pituitary remodeling in PSMA-negative prostate cancer.

Testis-derived radiomics demonstrated some of the strongest and most consistent predictive performance across CT-based, PET-based, and multimodal fusion pipelines. In the final analysis, the strongest TESTIS_CT + clinical model achieved an AUC of 0.729 (95% CI: 0.619–0.834), while the strongest TESTIS_PET + clinical model achieved an AUC of 0.733 (95% CI: 0.630–0.826). Moreover, the highest-performing overall multimodal fusion model combined TESTIS_CT and TESTIS_PET radiomics with clinical variables and achieved an AUC of 0.758 (95% CI: 0.653–0.849). Key radiomic biomarkers included HU standard deviation, GLCM Contrast, GLCM Autocorrelation, and GLSZM entropy-related features, reflecting tissue heterogeneity, structural irregularity, and gray-level nonuniformity. SHAP analysis further demonstrated that TESTIS-derived radiomic biomarkers consistently contributed among the strongest predictive features across both standalone and fusion models. Biologically, these findings align with the central role of testicular androgen production in sustaining androgen receptor signaling and prostate cancer progression. Chronic androgen deprivation therapy and endocrine adaptation may induce subtle structural remodeling, altered tissue density, fibrosis, or vascular heterogeneity within the testes that become detectable through radiomic analysis. The recurrent selection of TESTIS-derived biomarkers across independent pipelines suggests that testicular imaging phenotypes may reflect systemic hormonal adaptation related to recurrent or treatment-resistant disease rather than direct tumor involvement. Nevertheless, these interpretations remain exploratory, and future studies integrating testosterone levels, endocrine biomarkers, treatment response data, and longitudinal imaging will be required to clarify the biological basis and clinical significance of testicular radiomic heterogeneity in PSMA-negative prostate cancer [7].

The thyroid gland also demonstrated complementary predictive value, particularly within PET-based and clinically integrated models. In the final analysis, the strongest THYROID_PET + clinical model achieved an AUC of 0.678 (95% CI: 0.546–0.791), while THYROID_CT + clinical models achieved an AUC of 0.642 (95% CI: 0.523–0.751). Relevant radiomic biomarkers included GLSZM SizeZoneNonUniformity, first-order entropy, GLCM Autocorrelation, and texture heterogeneity features related to metabolic variability and signal complexity. Although thyroid-based models did not achieve the same level of performance as TESTIS- or HYPO_PIT-based pipelines, they demonstrated a moderate predictive contribution and relatively balanced specificity profiles. Biologically, these imaging patterns may reflect altered thyroid hormone regulation, vascular remodeling, or systemic inflammatory responses related to androgen deprivation therapy and endocrine adaptation. Experimental evidence suggests that thyroid hormones may enhance androgen signaling through both genomic and non-genomic pathways, including activation of androgen-related transcriptional programs and MAPK/ERK signaling cascades involved in prostate cancer progression [21]. Accordingly, increased texture entropy or heterogeneous uptake within the thyroid may represent indirect imaging correlates of systemic hormonal disequilibrium rather than organ-specific pathology. Nevertheless, these biological interpretations remain speculative, and future studies integrating thyroid function biomarkers, endocrine laboratory testing, and longitudinal imaging will be required to determine whether thyroid radiomic phenotypes have clinically meaningful associations with progression risk in PSMA-negative prostate cancer.

Integrating findings across endocrine organs suggests that systemic endocrine remodeling may leave quantifiable imaging signatures within hormonally active normal tissues. Across multiple predictive pipelines, recurrent radiomic biomarkers such as autocorrelation, entropy-related features, HU standard deviation, and gray-level nonuniformity were repeatedly related to progression prediction, indicating shared patterns of structural and metabolic heterogeneity. CT-based radiomics generally demonstrated greater structural stability and specificity, whereas PET-based models showed relatively higher sensitivity but appeared more vulnerable to metabolic noise and physiologic uptake variability. Importantly, multimodal fusion models integrating CT radiomics, PET radiomics, and clinical variables consistently achieved the strongest overall predictive performance, while true CT + PET fusion without clinical integration demonstrated substantially lower discrimination. These findings suggest that endocrine organ radiomics may provide complementary system-level information that contextualizes conventional clinicopathologic variables rather than functioning as independent predictive biomarkers.

A major observation of this study was the consistently strong performance of clinical variables across nearly all predictive pipelines. The strongest clinical-only configuration achieved an AUC of 0.727 (95% CI: 0.618–0.833), demonstrating that conventional clinicopathologic predictors retained substantial prognostic value in PSMA-negative prostate cancer. Dominant clinical contributors identified through SHAP analysis included Treatment Category, T Category, pN stage, Site of Lesion, and Primary Treatment, emphasizing the importance of tumor burden, treatment history, and disease stage in recurrence prediction. Although multimodal fusion models achieved slightly higher discrimination performance, DeLong analysis demonstrated that the incremental improvement beyond the clinical-only baseline remained modest and statistically nonsignificant. These findings highlight that the primary contribution of endocrine organ radiomics may lie in complementing and refining established clinical information rather than replacing it.

Biologically, the associations identified in this study should be interpreted cautiously and considered hypothesis-generating. Radiomic biomarkers reflect image-derived structural and textural variation rather than direct hormonal, metabolic, or molecular activity. Although prior biological evidence supports potential links between androgen signaling, adrenal steroidogenesis, thyroid hormone regulation, and endocrine axis remodeling in prostate cancer progression [19,20,21], the present study did not include endocrine laboratory biomarkers, pituitary MRI, or radiogenomic validation. Accordingly, the proposed endocrine-related mechanisms remain indirect and exploratory. Future studies integrating comprehensive hormonal profiling, including TSH, free T_4_/T_3_, cortisol, DHEA-S, LH, FSH, testosterone, and GnRH-related treatment data, together with external multi-center validation and longitudinal imaging, will be necessary to determine whether endocrine organ radiomic phenotypes truly reflect biologically meaningful systemic adaptation related to progression and treatment resistance in PSMA-negative prostate cancer.

## 5. Limitations

This study has several important limitations: First, it was a retrospective single-center analysis with a relatively small cohort of PSMA-negative patients, limiting statistical power and generalizability. Accordingly, the findings should be interpreted as exploratory and hypothesis-generating rather than definitive. Although multimodal fusion models numerically improved discrimination performance relative to the clinical-only baseline, these improvements did not reach statistical significance in DeLong analysis, likely reflecting both the modest incremental value of endocrine organ radiomics and the limited statistical power of the cohort. In addition, the absence of external validation and the relatively small sample size contributed to wide confidence intervals across several model configurations.

Manual segmentation of small endocrine structures on low-dose CT may introduce observer variability, partial-volume effects, and segmentation uncertainty, particularly within the hypothalamus–pituitary region. Interobserver reproducibility was not formally assessed. Furthermore, imaging alone cannot reliably identify subclinical endocrine abnormalities, and systematic chart reviews for thyroid disease, thyroid medication use, thyroidectomy, adrenal nodules, or other endocrine disorders were not performed. These factors may have introduced unrecognized biologic and imaging confounders.

Several additional technical and biological limitations should also be considered. The PET signal within normal endocrine and brain tissues is relatively low and partially influenced by physiologic blood pool activity, while low-dose CT provides limited soft-tissue contrast, increasing susceptibility to imaging noise and unstable radiomic measurements despite standardized preprocessing and strict fold-specific validation procedures. Moreover, radiomic biomarkers capture image-derived structural and textural variation rather than direct hormonal or molecular activity. Therefore, the proposed biological interpretation of endocrine organ radiomics as surrogate markers of systemic endocrine remodeling remains indirect and unvalidated. Follow-up imaging was also not protocolized, raising the possibility of lead-time bias, while post-imaging treatment heterogeneity may have influenced progression outcomes despite exclusion of post-imaging treatment variables from predictive modeling to avoid temporal data leakage. Future studies should incorporate external multi-center validation cohorts, endocrine laboratory biomarkers, radiogenomic analysis, interobserver reproducibility testing, and standardized imaging protocols to determine the biological and clinical robustness of these findings.

## 6. Conclusions

This study demonstrates that radiomic profiling of normal endocrine organs may reveal systemic imaging signatures related to clinical progression in PSMA-negative prostate cancer. Multimodal models integrating CT radiomics, PET radiomics, and clinical variables achieved the strongest overall predictive performance, with TESTIS-derived radiomic biomarkers consistently contributing across the highest-performing pipelines. Nevertheless, the incremental improvement beyond conventional clinical variables remained modest and did not reach statistical significance, suggesting that endocrine organ radiomics currently provide complementary rather than standalone predictive value.

The findings support the emerging concept that systemic tumor–host interactions and endocrine adaptation may manifest as subtle imaging phenotypes within hormonally active organs, including the adrenal glands, the hypothalamus–pituitary complex, testes, and thyroid. Although the biological interpretation of these imaging signatures remains exploratory, this multi-organ radiomics framework provides a novel non-invasive approach for investigating systemic disease biology in patients with imaging-negative but clinically progressive prostate cancer. Future multi-center studies integrating endocrine biomarkers, radiogenomics, and longitudinal imaging will be essential to determine whether endocrine organ radiomics can provide clinically meaningful value for risk stratification and precision oncology in PSMA-negative prostate cancer.

## Figures and Tables

**Table 1:** Performance of the strongest single-organ CT- and PET-based models with and without clinical integration. Abbreviations: GB, gradient boosting; HGB, histogram-based gradient boosting; Log Reg, logistic regression.

Category	Combination	FS_Model	ML_Model	AUC_95%CI	Accuracy	Sensitivity	Specificity	Brier	Log Loss
CT + Clinical	ADRENAL	LASSO	Random Forest	0.700 (0.590–0.809)	0.681	0.585	0.816	0.233	1.051
CT + Clinical	HYPO_PIT	RFE	HGB	0.709 (0.591–0.813)	0.692	0.774	0.579	0.24	0.705
CT + Clinical	TESTIS	SelectKBest	SVM	0.729 (0.619–0.834)	0.67	0.623	0.737	0.206	0.604
CT + Clinical	THYROID	SelectKBest	HGB	0.642 (0.523–0.751)	0.648	0.736	0.526	0.273	0.848
CT-Only	ADRENA	SelectKBest	SVM	0.539 (0.423–0.657)	0.571	0.453	0.737	0.244	0.681
CT-Only	HYPO_PIT	LASSO	Log Reg	0.558 (0.433–0.687)	0.637	0.755	0.474	0.273	0.766
CT-Only	TESTIS	SelectKBest	Random Forest	0.636 (0.515–0.746)	0.604	0.491	0.763	0.24	0.681
CT-Only	THYROID	RFE	Random Forest	0.500 (0.382–0.627)	0.527	0.453	0.632	0.279	0.776
PET + Clinical	ADRENAL	SelectKBest	HGB	0.695 (0.593–0.798)	0.637	0.528	0.789	0.25	0.747
PET + Clinical	HYPO_PIT	LASSO	HGB	0.726 (0.616–0.830)	0.703	0.717	0.684	0.233	0.72
PET + Clinical	TESTIS	SelectKBest	HGB	0.733 (0.630–0.826)	0.692	0.679	0.711	0.221	0.679
PET + Clinical	THYROID	SelectKBest	SVM	0.678 (0.546–0.791)	0.703	0.698	0.711	0.222	0.64
PET-Only	ADRENA	RFE	HGB	0.585 (0.468–0.705)	0.549	0.377	0.789	0.274	0.786
PET-Only	HYPO_PIT	LASSO	GB	0.594 (0.476–0.714)	0.626	0.717	0.5	0.305	0.968
PET-Only	TESTIS	RFE	HGB	0.597 (0.480–0.713)	0.604	0.604	0.605	0.27	0.791
PET-Only	THYROID	RFE	HGB	0.710 (0.587–0.827)	0.714	0.868	0.5	0.219	0.67

**Table 2:** Performance of the strongest multi-organ and multimodal fusion models for prediction. Abbreviations: GB, gradient boosting; HGB, histogram-based gradient boosting; Log Reg, logistic regression.

Category	Combination	FS_Model	ML_Model	AUC_95CI	Accuracy	Sensitivity	Specificity	Brier	Log Loss
Multi-Organ CT-Only	ADRENAL + HYPO_PIT + TESTIS + THYROID	SelectKBest	Log Reg	0.597 (0.474–0.715)	0.549	0.321	0.868	0.25	0.695
Multi-Organ CT-Only	ADRENAL + TESTIS	SelectKBest	Log Reg	0.619 (0.496–0.730)	0.582	0.415	0.816	0.24	0.671
Multi-Organ CT-Only	ADRENAL + TESTIS + THYROID	SelectKBest	Log Reg	0.619 (0.496–0.730)	0.582	0.415	0.816	0.24	0.671
Multi-Organ PET-Only	ADRENAL + HYPO_PIT + TESTIS + THYROID	RFE	GB	0.605 (0.486–0.728)	0.615	0.604	0.632	0.308	0.974
Multi-Organ PET-Only	ADRENAL + TESTIS + THYROID	SelectKBest	HGB	0.62 (0.509–0.750)	0.648	0.604	0.711	0.275	0.801
Multi-Organ PET-Only	ADRENAL_PET + THYROID_PET	RFE	Log Reg	0.678 (0.553–0.786)	0.692	0.679	0.711	0.235	0.681
CT + PET + Clinical	ADRENAL_CT + ADRENAL_PET	LASSO	Random Forest	0.679 (0.565–0.794)	0.692	0.698	0.684	0.237	0.711
CT + PET + Clinical	HYPO_PIT_CT + HYPO_PIT_PET	LASSO	HGB	0.725 (0.622–0.819)	0.681	0.66	0.711	0.238	0.7
CT + PET + clinical fusion	TESTIS_CT + TESTIS_PET	RFE	GB	0.758 (0.653–0.849)	0.703	0.604	0.842	0.254	0.995
CT + PET + Clinical	THYROID_CT + THYROID_PET	SelectKBest	SVM	0.678 (0.546–0.791)	0.703	0.698	0.711	0.222	0.64
CT + PET	ADRENAL_CT + ADRENAL_PET	LASSO	SVM	0.563 (0.451–0.679)	0.571	0.509	0.658	0.237	0.666
CT + PET	HYPO_PIT_CT + HYPO_PIT_PET	LASSO	Log Reg	0.547 (0.418–0.672)	0.626	0.868	0.289	0.29	0.804
CT + PET	TESTIS_CT + TESTIS_PET	SelectKBest	Random Forest	0.636 (0.515–0.746)	0.604	0.491	0.763	0.24	0.681
CT + PET	THYROID_CT + THYROID_PET	RFE	HGB	0.662 (0.540–0.771)	0.659	0.585	0.763	0.249	0.721

**Table 3:** Performance of the strongest machine learning classifiers for prediction. Abbreviations: HGB, histogram-based gradient boosting; Log Reg, logistic regression; SVM, support vector machine.

ML_Model	Category	Combination	FS_Model	AUC_95CI	Accuracy	Sensitivity	Specificity	Brier	Log Loss
Gradient Boosting	CT + PET + Clinical	TESTIS_CT + TESTIS_PET	RFE	0.758 (0.653–0.849)	0.703	0.604	0.842	0.254	0.995
HGB	CT + PET + Clinical	TESTIS_CT + TESTIS_PET	RFE	0.757 (0.646–0.857)	0.747	0.792	0.684	0.211	0.701
Log Reg	PET + Clinical	ADRENAL_PET	RFE	0.687 (0.565–0.803)	0.681	0.623	0.763	0.24	0.701
Random Forest	CT + PET + Clinical	TESTIS_CT + TESTIS_PET	RFE	0.751 (0.644–0.843)	0.725	0.755	0.684	0.208	0.632
SVM	CT + Clinical	TESTIS_CT	SelectKBest	0.729 (0.619–0.834)	0.67	0.623	0.737	0.206	0.604

**Table 4:** Performance of the strongest feature selection approaches for prediction. Abbreviations: GB: gradient boosting, RFE, recursive feature elimination; HGB, histogram-based gradient boosting.

FS_Model	Category	Combination	ML_Model	AUC_95CI	Accuracy	Sensitivity	Specificity	Brier	Log Loss
LASSO	CT + PET + Clinical	TESTIS_CT + TESTIS_PET	HGB	0.742 (0.636–0.836)	0.714	0.755	0.658	0.228	0.745
RFE	CT + PET + Clinical	TESTIS_CT + TESTIS_PET	GB	0.758 (0.653–0.849)	0.703	0.604	0.842	0.254	0.995
SelectKBest	PET + Clinical	TESTIS_PET	HGB	0.733 (0.630–0.826)	0.692	0.679	0.711	0.221	0.679

## Data Availability

The data supporting the findings of this study are not publicly available due to privacy and ethical restrictions but may be available from the corresponding author upon reasonable request.
